# An unusually long Rift valley fever inter-epizootic period in Zambia: Evidence for enzootic virus circulation and risk for disease outbreak

**DOI:** 10.1371/journal.pntd.0010420

**Published:** 2022-06-02

**Authors:** Herman M. Chambaro, Kazuyo Hirose, Michihito Sasaki, Brigadier Libanda, Yona Sinkala, Paul Fandamu, Walter Muleya, Fredrick Banda, Joseph Chizimu, David Squarre, Misheck Shawa, Yongjin Qiu, Hayato Harima, Yuki Eshita, Edgar Simulundu, Hirofumi Sawa, Yasuko Orba

**Affiliations:** 1 International Institute for Zoonosis Control, Hokkaido University, Sapporo, Japan; 2 Virology Unit, Central Veterinary Research Institute, Lusaka, Zambia; 3 Ministry of Fisheries and Livestock, Lusaka, Zambia; 4 Japan Space Systems, Tokyo, Japan; 5 The University of Edinburgh, School of Geosciences, Edinburgh, Scotland, United Kingdom; 6 The University of Zambia, School of Veterinary Medicine, Lusaka, Zambia; 7 Ministry of Health, Lusaka, Zambia; 8 The University of Edinburgh, Royal Dick School of Veterinary Studies, Edinburgh, Scotland, United Kingdom; 9 Macha Research Trust, Choma, Zambia; 10 Global Virus Network, Baltimore, Maryland, United States of America; 11 International Collaboration Unit, International Institute for Zoonosis Control, Hokkaido University, Sapporo, Japan; 12 One Health Research Center, Hokkaido University, Sapporo, Japan; University of California Irvine, UNITED STATES

## Abstract

Rift valley fever (RVF) is a mosquito-borne disease of animals and humans. Although RVF outbreaks are usually reported at 5-15-year intervals in sub-Saharan Africa, Zambia has experienced an unusually long inter-epizootic/-epidemic period of more than three decades. However, serological evidence of RVF virus (RVFV) infection in domestic ruminants during this period underscores the need for comprehensive investigation of the mechanisms of virus perpetuation and disease emergence. Mosquitoes (*n* = 16,778) captured from eight of the ten provinces of Zambia between April 2014 and May 2019 were pooled (*n* = 961) and screened for RVFV genome by a pan-phlebo RT-PCR assay. *Aedes* mosquito pools (*n* = 85) were further screened by nested RT-PCR assay. Sera from sheep (*n* = 13), goats (*n* = 259) and wild ungulates (*n* = 285) were screened for RVFV antibodies by ELISA while genome detection in pooled sera (*n* = 276) from domestic (*n* = 248) and wild ungulates (*n* = 37) was performed by real-time RT-PCR assay. To examine the association between the long inter-epizootic period and climatic variables, we examined El Niño-Southern Oscillation indices, precipitation anomalies, and normalized difference vegetation index. We then derived RVF risk maps by exploring climatic variables that would favor emergence of primary RVFV vectors. While no RVFV genome could be detected in pooled mosquito and serum samples, seroprevalence was significantly high (OR = 8.13, 95% CI [4.63–14.25]) in wild ungulates (33.7%; 96/285) compared to domestic ruminants (5.6%; 16/272). Retrospective analysis of RVF epizootics in Zambia showed a positive correlation between anomalous precipitation (La Niña) and disease emergence. On risk mapping, whilst northern and eastern parts of the country were at high risk, domestic ruminant population density was low (< 21 animals/km^2^) in these areas compared to low risk areas (>21 animals/km^2^). Besides evidence of silent circulation of RVFV and the risk of disease emergence in some areas, wildlife may play a role in the maintenance of RVFV in Zambia.

## Author summary

Rift valley fever (RVF) is an important mosquito borne disease of humans and ruminants. RVF is endemic in sub-Saharan Africa where disease outbreaks are frequently reported in humans and animals. For RVF outbreaks to occur, there has to be presence of the host (animals or humans) and favorable climatic conditions that favor emergence of RVF virus vectors (mosquitoes). Understanding this complex interaction enables us to predict future disease outbreaks and map RVF high risk areas. Above normal rainfall and floods will usually trigger RVF outbreaks. In this study, we conducted surveillance for RVF in mosquitoes, domestic and wild ruminants. We then examined climatic conditions that may have been responsible for past RVF outbreaks and the unusually (>30 years) long inter-epizootic period. Finally, we derived RVF risk maps for Zambia using a combination of climatic variables. We found evidence of RVF infection in both wild and domestic ruminants while there was a positive correlation between past RVF outbreaks and above-normal rainfall. On risk mapping, we found favorable conditions (i.e. high rainfall and risk of flooding) for RVF emergence in northern and eastern parts of the country. Studies like this are important in planning surveillance and disease control programs.

## Introduction

Rift Valley fever (RVF), caused by the RVF virus (RVFV; family *Phenuiviridae*, genus *phlebovirus*), is an emerging arthropod-borne zoonosis that is primarily transmitted to animals and humans by mosquitoes [1,2]. RVF is listed by the World Organization for Animal Health and is considered a priority disease by the World Health Organization [3]. RVFV is vectored by over 53 species of mosquitoes from eight genera, although only *Neomelaniconion* and *Aedimorphus* mosquitoes are considered as primary vectors [4]. In ruminants, the disease presents as generalized fever with widespread abortions while in humans, although usually self-limiting, 1% of all affected individuals develop hemorrhagic fever or encephalitis [5].

RVF was first reported in Kenya in 1931 following mortalities and abortions in sheep [1,6]. Since then, major epizootics/epidemics have been reported in a number of African countries [7]. Usually, disease outbreaks occur at irregular intervals of 5–15 years in wet regions and 25 years in the drier areas [5,8]. Epizootics/epidemics are associated with periods of excessive rainfall and persistent flooding of geomorphic depressions, also known as dambos in Africa. Three-months of sustained above-normal rainfall has been reported to trigger RVF outbreaks [9]. Floodwater-breeding *Aedes* mosquitoes (subgenera; *Aedimorphus* and *Neomelaniconion*) are responsible for virus maintenance through transovarial transmission [10]. *Aedimorphus* and *Neomelaniconion* mosquitoes lay eggs in the mud at the edges of dambos which may survive long periods of drought [11]. The flooding of dambos during periods of excessive or anomalous rainfall results in the hatching and emergence of large numbers of infected *Aedes* mosquitoes [5]. Initial transmission of RVFV to susceptible domestic and wild ruminants and subsequent recruitment of ‘bridge’ mosquitoes (*Culex*, *Anopheles etc*.*)* results in sustained, widespread infection. Conversely, during the inter-epizootic/-epidemic period when conditions are not favorable, there is limited emergence of infected Aedine mosquitoes which results in restricted transmission of the virus to susceptible domestic and wild ruminants [12–15]. Due to the difficulties associated with identifying single or isolated cases of RVF in the inter-epizootic/-epidemic period, there is usually ‘silent’ circulation of the virus where infected animals play an important role in pathogen survival through horizontal transmission [16].

Although the role of domestic ruminants in the epidemiology of RVF is well clarified, there is limited information on the role wildlife play in the maintenance of RVFV during the inter-epizootic/-epidemic period [2,17]. In Zambia, no studies have been conducted to clarify the role of wildlife in the epidemiology of RVF. Reports from other African countries suggests that wildlife might play an important role in the maintenance and transmission of RVFV [2,7,12,13]. Historically, Zambia has experienced a number of RVF epizootics that have been associated with fatal disease in humans [18,19]. The first case of RVF was reported in 1974 in cattle and sheep from Central Province (Chisamba District) and subsequently in Southern (Mazabuka District) and parts of the Copperbelt Province [20]. In 1976 and 1978, there was recurrence of the disease in Chisamba District [20] followed by another outbreak in 1985 in the same area and Mazabuka District [21,22]. Even though no further outbreaks have been reported since then, subsequent serosurveillance studies intimate presence of, and ‘silent’ circulation of the RVFV in domestic ruminants in Zambia [20–29].

While the reasons for the unusually long inter-epizootic/-epidemic period are largely speculative, emergence of RVF in South Africa, Tanzania and Kenya was linked to increased rainfall and vegetation density [9,10,30]. In Zambia (Central Province; Mumbwa District), Davies et al., [27] found a positive correlation between increased vegetation and RVFV seropositivity in cattle. In recent years, a number of climate variables have been proposed as predictors for RVF emergence [4,9,10,31]. The El Niño/Southern Oscillation (ENSO) phenomenon, a variation in sea surface temperature (SST) and atmospheric pressure (Southern Oscillation) across the equatorial Pacific Ocean influences global inter-annual climate variability through the so-called ‘teleconnections’ [32,33]. El Niño (La Niña) is characterized by a five consecutive 3-months running mean of SST anomalies in the equatorial eastern-central Pacific Ocean (Niño 3.4) region that are above +0.5°C (-0.5°C). El Niño (La Niña) reduces (increases) precipitation over the south-eastern Africa, while increasing (decreasing) precipitation over the northeastern tropical region [34–36]. ENSO indices i.e., Southern Oscillation Index (SOI), NINO 3.4 SST and outgoing longwave radiation (OLR) are used to predict above normal rainfall and RVF emergence [9]. Positive SOI (> 1.0), OLR (> 1.0 w/m^2^) and negative NINO 3.4 SST (< -0.5) anomalies are associated with increased precipitation in Southern Africa [37]. Equally, measurement of vegetation greenness through satellite imaging, expressed as a normalized difference vegetation index (NDVI), is used as a proxy for estimating precipitation. NDVI values close to zero indicate bare soil, and high values indicate sparse to dense vegetation. Furthermore, during periods of excessive precipitation, increased soil moisture content has been correlated with flooding of dambos and emergence of RVF epizootics [10,38].

In this study, we conducted surveillance for RVFV in mosquitoes, domestic and wild ungulates in Zambia. We then examined climatic variables that were likely responsible for past RVF outbreaks and the unusually long inter-epizootic/-epidemic period. Lastly, we derived RVF risk maps by analyzing various climatic indices. We anticipate that this information will be useful for planning and implementation of surveillance and disease control programs in Zambia.

## Materials and methods

### Ethics statement

The study was approved by the Ministry of Fisheries and Livestock, Government of the Republic of Zambia as part of the continued surveillance for zoonoses. Blood samples from free-ranging wildlife were obtained with permission (TJ/NPW/8/27/1) from the Department of National Parks and Wildlife, under the Ministry of Tourism and Arts, Government of the Republic of Zambia.

### Study area

**Fig 1:**
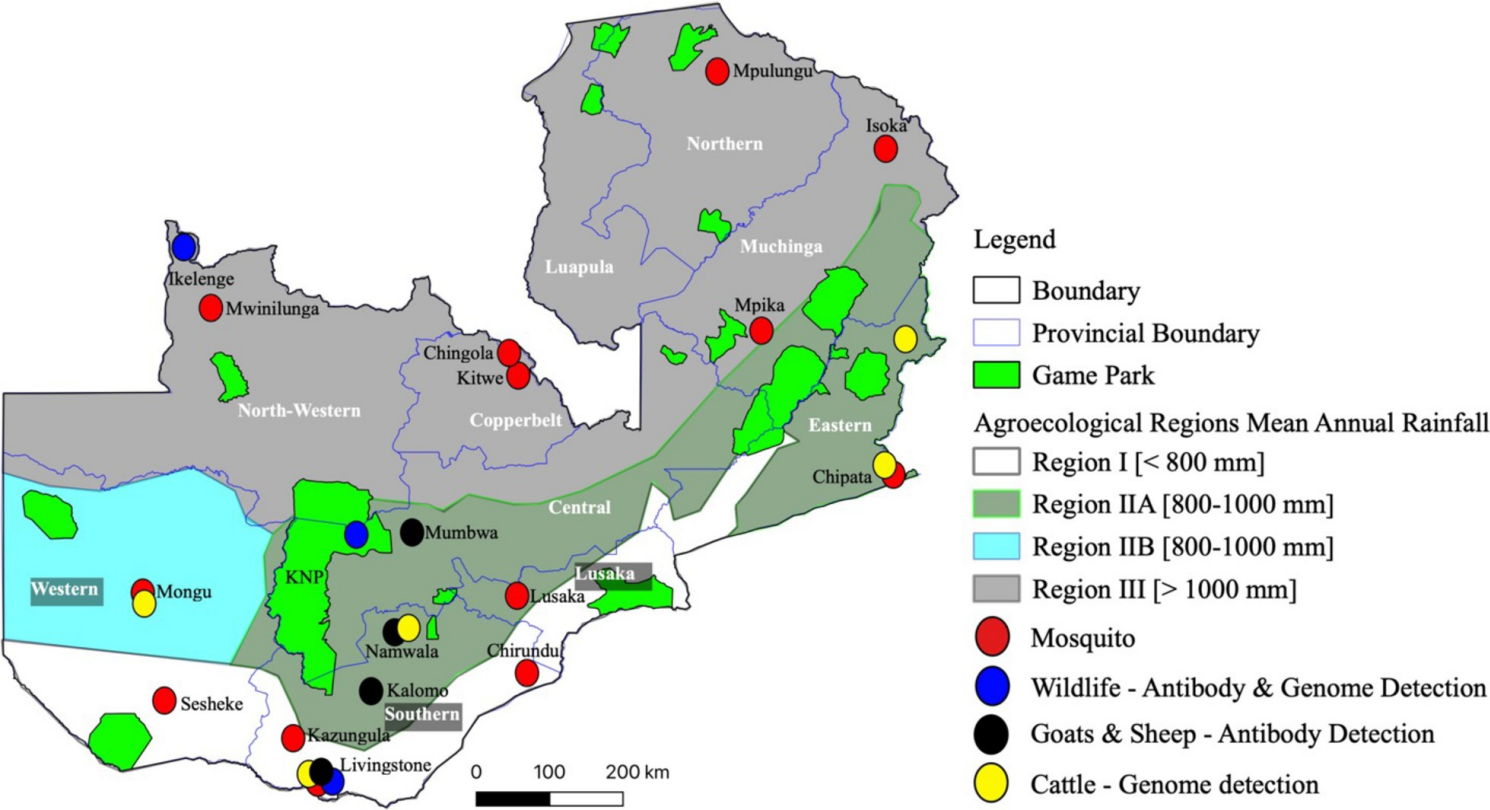
Agroecological regions and location of sample collection areas by district. Shapefile republished from DIVA-GIS database (https://www.diva-gis.org/) under a CC BY license of Global Administrative Areas (GADM), copyright 2018.

Zambia has a total landmass of approximately 752,614 km^2^ and is located in south-central Africa between latitudes 8° and 18° south and longitudes 22° and 34° east. The climate is characterized by three seasons; cool-dry season (May to August), hot-dry season (August to November) and hot-wet season (November to April) [39]. The intertropical convergence zone (ITCZ) of northeasterly and southeasterly trade winds, and ENSO influence the interannual climate variability in Zambia and other sub-Saharan countries [40–43]. During the rainy season, the December-January-February (DJF) period accounts for over 80% of total precipitation [44]. On the account of mean annual precipitation, Zambia is broadly divided into three agroecological regions (Fig 1).

Region I is drier with a mean annual rainfall of less 800 mm, while region II receives between 800 to 1000 mm of rain. Region III has a mean annual rainfall of 1000 to 1500 mm.

### Mosquito samples

Mosquitoes (*n* = 1,553) were trapped in Mosi-oa-Tunya National Park in Southern Province after the rainy season in May 2019 using the CO_2_-baited CDC light traps (John W. Hock Co., Gainesville, FL, USA). Traps were set at a height of approximately 1.5 meters (effective height) from the ground for five consecutive nights (from 3PM to 10AM) at different locations near water ponds and in abandoned buildings within the national park. Captured mosquitoes were killed by freezing at -20°C in a mobile freezer and later transferred to -80°C until further analysis. Mosquitoes were then sorted by sex and species on ice packs using morphological referencing keys of African mosquitoes [45]. Further confirmation of some mosquito species was achieved through PCR and sequencing of the *cytochrome oxidase I* (COI) gene [46]. Female mosquitoes were pooled (1~40) by species, and homogenized in minimum essential medium supplemented with 2% foetal bovine serum as previously described [47]. The homogenate was clarified by centrifugation and RNA extracted from the supernatant using the Direct-Zol RNA kit (Zymo Research, CA, USA) according to the manufacturer’s recommendation. Additionally, archived RNA (*n* = 15,225) from mosquitoes previously captured in Eastern, Copperbelt, Lusaka, Muchinga, Northern, North-Western, Southern, and Western provinces between April 2014 and December 2018 [47–49] were also analyzed in this study. In summary, a total of 16,778 mosquito samples from eight of the ten provinces (Fig 1) were pooled (*n* = 961) and analyzed. Notably, due to various logistical challenges, mosquito sampling could not be standardized across various regions.

### Serum samples

A total of 635 blood samples were randomly collected from unvaccinated, apparently healthy domestic ruminants in Southern (*n* = 335) and Western Province (*n* = 300) between August 2018 and May 2019 (Fig 1 and Table 1). Of these, 446 samples were collected from cattle while 189 were collected from sheep (*n* = 13) and goats (*n* = 176). In brief, blood was collected from the jugular vein from each animal into plain tubes, allowed to clot, and serum was separated by centrifugation at 1,500 x g for 5 minutes. Sera was stored at -80°C until analysis. Additionally, Stored sera (*n* = 740) collected from asymptomatic, unvaccinated domestic (*n* = 560) [50–52] and wild ungulates (*n* = 180) [53,54] between December 2016 and June 2017 were analyzed in this study (Fig 1 and Table 1). In total, 1,375 serum samples were used for subsequent analysis. For RVFV genome detection, serum samples were pooled (~1/5; *n* = 276) by species and geographical origin, and RNA was extracted using the QIAamp Viral RNA Mini Kit (Qiagen, Hilden, Germany) according to manufacturer’s recommendation. For antibody detection, due to resource limitation, individual serum samples (*n* = 557) collected from sheep (*n* = 13), goats (*n* = 259) and wild ruminants (*n* = 285) in selected districts of Southern, North-Western and Central Province were used for analysis (Fig 1).

**Table 1:** Composition of sera from domestic and wild ruminants used for RVFV genome detection.

Province	District/Area	Year	Month	Species	No. Samples	No. Pools
Southern	Livingstone	2019	April	Cattle	104	21
	Livingstone	2019	April	Goats	99	20
	MNP	2017	August	Buffaloes	30	6
	Namwala	2018	May	Cattle	132	26
North-Western	Ikelenge	2016	December	Buffalo	5	1
Central	KNP	2017	June	Impala	98	20
	KNP	2017	June	Hartebeest	29	6
	KNP	2017	June	Warthog	18	4
Eastern	Chipata	2017	May	Cattle	350	70
	Lundazi	2018	May	Cattle	210	42
Western	Mongu	2018	August	Cattle	210	42
	Mongu	2018	August	Goats	77	15
	Mongu	2018	August	Sheep	13	3
Total					1375	276

MNP., Mosi-oa-Tunya National Park; KNP., Kafue National Park

### RVFV antibody detection

Serologic analysis for RVFV antibodies was performed using the ID Screen Rift Valley Fever Competition Multi-species ELISA assay (IDVet, Grabels, France) which is based on the recombinant nucleoprotein of RVFV. The ID screen RVF Competition Multi-species ELISA targets both IgG and IgM.

### RVFV genome detection

#### Mosquito samples

To detect RVFV in pooled mosquito samples (*n* = 961), we designed Pan-Phlebo RT-PCR primers (i.e., sense primer-L-2779F CARCATGGWGGTYTDAGRGARATCTA and antisense Primer-L-3287R TGCARKATKCCYTGCATCATHCCWG) in primer3 software [55] that target the partial (~500 bp) L segment of phleboviruses. Screening for the RVFV genome was carried out using the One Step PrimeScript RT-PCR kit (Takara, Shiga, Japan) in a 15 μl reaction mix containing; 0.6 μl of Takara PrimeScript Enzyme Mix, 7.5 μl of 2X 1-step buffer, 1 μM of each of the forward and reverse primer, and 1 μl of RNA template. The samples were incubated for 30 minutes at 50°C and 2 minutes at 94°C followed by amplification of 43 cycles consisting of 30 seconds at 94°C, 30 seconds at 52°C and final extension at 72°C for 5 minutes. The RT-PCR assay detection limit was 20 copies of RVFV RNA (S1 Fig). Furthermore, as a consequence of the reported long term maintenance of RVFV in Aedine mosquitoes [2], all *Aedes* mosquito pools (*n* = 85) negative for the RVFV genome on the Pan-Phlebo RT-PCR assay were further screened using a sensitive nested RT-PCR targeting the partial (~374 bp) M segment of the RVFV genome [56].

#### Serum samples

For pooled serum samples (*n* = 276; Table 1), the extracted RNA was screened for the RVFV genome using the real-time RT-PCR assay [57] using the Luna Universal Probe One-Step RT-qPCR Kit (New England Biolabs, Ipswich, England).

### Statistical analysis

Prevalence of RVFV antibodies was calculated in EpiTools epidemiological calculators [58] using confidence limits for apparent prevalence and true prevalence estimates assuming an imperfect test [59] with a reported test sensitivity and specificity of 100% [60]. Measures of association were computed using epi.2by2 function of the package “epiR” version 2.0.33 [61].

### Analysis of past RVF outbreaks

To assess conditions that may have been responsible for the emergence of past RVF outbreaks in Chisamba, Mazabuka and Chingola districts in Zambia, we analyzed ENSO indices retrieved from the National Oceanic and Atmospheric Administration (NOAA) National Centers for Environmental Information (https://www.ncdc.noaa.gov). We tracked the phase and amplitude of SOI, OLR and Niño 3.4 anomalies for the period January 1, 1973 to December 31, 2019 as previously described [9,31]. Precipitation anomalies for the period January 1, 1981 to December 31, 2019 were obtained from the Climate Hazards Group InfraRed Precipitation with Station (CHIRPS) data version 2 [62] while precipitation data prior to 1981 (1970–1980) was obtained from the Climate Research Unit [63]. CHRIPS data with a resolution of 0.05° x 0.05° (~5.5 km) extends from 1981 to present while CRU data with a low resolution (0.5 x 0.5; 55 km) spans from 1901 to 2018 [63]. Furthermore, standardized precipitation-evaporation index (SPEI) datasets (January 1, 1973 to December 31, 1990) for Chisamba, Mazabuka and Chingola districts were retrieved from the Global SPEI database (https://spei.csic.es/database). SPEI, a drought monitoring tool, is a derivative of precipitation and temperature in the form of simple water balance [64].

### Mapping RVF risk areas

#### NDVI Maps

RVF risk maps were generated using previously described criteria [31,65–68]. Briefly, RVF emergence is a result of the complex interaction of the host, vector and environment. Increased RVF activity is triggered by persistent above-normal rainfall and floods [9,27,31,65–67] which favor the breeding of transovarially infected *Aedes neomelaniconion* and/or *Aedimorphus* mosquitoes [66]. Three months of sustained above normal rainfall and increased NDVI are indicative of probable RVF outbreaks [66,69]. Thus, firstly, to assess seasonal changes in potential mosquito breeding habitats in Zambia, we retrieved (United States Geological Survey data hub; https://earthexplorer.usgs.gov) and calculated the 19-year (2000–2019) mean NDVI (NDVI = [NIR-RED]/[NIR+Red]) in QGIS software (http://www.qgis.org) at the beginning (November) and end (March) of the rainy season. NDVI data, MOD13A3, used in the analysis are monthly 1-kilometer spatial resolution gridded level 3 products acquired by the Terra Moderate Resolution Imaging Spectroradiometer (MODIS) satellite.

Secondly, we performed morphometric characterization of watersheds in QGIS (http://www.qgis.org) using void filled Shuttle Radar Topographic Mission (SRTM) Digital Elevation Model (DEM) data (https://earthexplorer.usgs.gov) with a spatial ground resolution of 30 m (1 arc-second). A 2 km watershed buffer representing probable catchment area was used to calculate riparian NDVI. Areas with dense vegetation representing probable mosquito breeding habitats were then calculated from the riparian NDVI.

#### Mean DJF precipitation and soil moisture content

To asses DJF precipitation patterns and areas with high likelihood of flooding, Tropical Rainfall Measuring Mission (TRMM) and Multi-Satellite Precipitation Analysis monthly gridded, (0.25° x 0.25°; ~ 25 km) 3B43 (version 7) products were used to estimate time-averaged (1998–2020) DJF precipitation over Zambia using Giovanni webtool (https://giovanni.gsfc.nasa.gov.) TRMM 3B43 monthly products are a derivative of the 3B42 hourly datasets created using TRMM-adjusted microwave-infrared precipitation rate (mm/hour) and root mean square precipitation-error estimates (https://disc.gsfc.nasa.gov). Additionally, National Aeronautics Space Association (NASA) Goddard Earth Science and Information Services Centre (GES DISC) Global Land Data Assimilation System (GLDAS) of 0.25° spatial resolution, which uses satellite and ground-based observations to generate flux data [70], was used to determine the mean (2000–2020) DJF soil moisture content in the near surface soil layer (0–10 cm) for Zambia. GLDAS version 2.1 soil moisture products, forced with a combination of model and observation data were processed in Giovanni (https://giovanni.gsfc.nasa.gov) and QGIS software.

#### Ruminant population density, serosurveillance data and RVF risk mapping

Domestic ruminant (i.e., cattle, goats, and sheep) population density (animals/km^2^) by district were calculated in QGIS software using the 2017/18 Livestock and Aquaculture Census Data provided by the Department of Veterinary Services in the Ministry of Fisheries and Livestock, Government of the Republic of Zambia. Furthermore, historic RVF outbreaks [19,21,28], past [20,22–28] and present serosurveillance data were modelled to the risk map.

### Detection of permanent and ephemeral waterbodies (Dambos)

To detect permanent and/or ephemeral water bodies that would potentially serve as breeding grounds for *Aedimorphus* and *Neomelaniconion* mosquitoes in Zambia, we analyzed Copernicus sentinel-1 datasets in interferometric wide swath (IW) acquisition mode and ground range detected (GRD) format for two selected areas in Monze and Chililabombwe districts. Monze District was selected as a representative district in a low rainfall (800–1000 mm; Region II) area with the highest domestic ruminant population density while Chililabombwe District was selected based on its location in a high rainfall (> 1000 mm; Region III) zone with a relatively high ruminant population density. Sentinel-1 data sets (2017 to 2020) at the beginning (October) and end (March) of the rain season were acquired from the European space association (ESA) data hub (https://scihub.copernicus.eu) and preprocessed using the Sentinel Application Platform (SNAP; https://step.esa.int) and QGIS software as previously described [71,72]. Initially, we applied a precise orbit state vector and removed thermal noise to reduce noise effects in the inter-sub-swath texture and normalize backscatter signal. Next, border noise was removed to eliminate radiometric artefacts and radiometric calibration was applied to convert digital pixel values to backscatter coefficient sigma nought. Range doppler terrain correction using SRTM 30 m (1 arc-second) resolution dataset was employed to compensate for distortions related to side-looking geometry. The backscatter coefficient was then logarithmically transformed to decibels (dB) and raster calculations were then performed in SNAP and QGIS software. A split-based global thresholding technique was used to construct a bimodal histogram [73]. Water body delineation was conducted as previously described [73,74].

## Results

### Diversity and distribution of captured mosquitoes

A total of 16,778 mosquitoes belonging to seven genera were captured from eight of the 10 provinces of Zambia (Tables 2 and S1). *Culex* (73.9%) was the most abundant species caught during the surveillance period. Western (50.2%) and Southern (30.3%) provinces accounted for most (80.5%) of the mosquitoes captured while the rest were captured in Lusaka (11.7%), Northern (2.3%), Muchinga (1.6%), Eastern (1.8%), Copperbelt (1.8%) and North-Western (0.3%) provinces. Species diversity, calculated using the Simpsons diversity index [75], was 0.43.

**Table 2:** Composition of mosquito species collected during the study period.

Genus	No. of Mosquitoes	No. of Pools Screened	Percent of Total (%)
*Aedeomya*	14	6	0.08
*Uranotaenia*	14	6	0.08
*Aedes*	346	85	2.02
*Coquillettidia*	524	60	3.12
*Mansonia*	905	58	5.40
*Anopheles*	2583	176	15.40
*Culex*	12392	570	73.86
Total	16778	961	100

Varying numbers of *Aedes* mosquitoes were captured in seven of the eight provinces (Tables 3 and S1). Southern Province accounted for 73.8% (251/340) of all *Aedes* mosquitoes while the rest were caught in Western (12.6%; 43/340), Lusaka (6.8%; 23/340), Northern (5.3%; 18/340), Muchinga (1.8%; 6/340), North-Western (1.2%; 4/340), and Eastern (0.3%; 1/340) provinces. No *Aedes* mosquitoes were captured on the Copperbelt Province. Furthermore, only 9.4% (32/340) of the *Aedes* mosquitoes were classified as primary RVF vectors of the *Neomelaniconion* (*Aedes mcintoshi*; *n* = 31) and *Aedimorphus* (*Aedes ochraceus*; *n* = 1) subgenus (Table 3). The rest were *Stegomyia* (84.1%; 286/340), *Fredwardsius* (0.59%; 2/340) and unidentified *Aedes* species (6.2%; 21/340). *Aedes mcintoshi* (*Neomelaniconion*) and *Aedes ochraceus* (*Aedimorphus)* were caught in Western (December and May) and Southern (December) provinces, respectively.

**Table 3:** Primary and secondary RVF vectors captured by province in Zambia.

Province	Primary vector		Secondary vector									
	*Aedes mcintoshi*	*Aedes ochraceus*	*Aedes aegypti*	*Aedes vittatus*	*Aedes* ^†^	*Aedeomya*	*Uranotaenia*	*Coquillettidia*	*Mansonia*	*Anopheles*	*Culex*	Total
Southern	0	1	234	1	15	2	0	0	78	124	4619	**5074**
Western	31	0	11	0	1	12	7	417	823	2425	4696	**8423**
Eastern	0	0	1	0	0	0	0	0	0	0	307	**308**
Northern	0	0	10	0	8	0	0	0	1	3	367	**389**
North-Western	0	0	3	1	0	0	2	0	0	3	40	**49**
Lusaka	0	0	23	0	0	0	0	0	2	2	1939	**1966**
Muchinga	0	0	6	0	0	0	5	107	1	26	130	**275**
Copperbelt	0	0	0	0	0	0	0	0	0	0	294	**294**
**Total**	**31**	**1**	**288**	**2**	**24**	**14**	**14**	**524**	**905**	**2,583**	**12,392**	**16,778**

^†^Unidentified *Aedes* species

### RVFV seroprevalence and genome detection

Antibodies (IgM and IgG) to RVFV were detected in 20.1% (112/557) of domestic and wild ruminants tested by ELISA (Table 4). The overall seroprevalence was 20.1% (95% CI [17.0–23.6]), although this was significantly high (OR = 8.13, 95% CI [4.63–14.25]) in wildlife (33.7%; 96/285) compared to domestic ruminants (5.9%; 16/272). The seroprevalence in buffaloes from Southern Province was significantly high (50%) compared to that in buffaloes from Central (35.3%, OR = 7.62, 95% CI [2.73–21.30]) and North-Western (24.1%; OR = 3.14 95% CI [1.03–9.55]) Province. Seroprevalence in Impala from Kafue National Park in Central Province was similar to that in hartebeest (46.9% Vs 43.9%), however, this was significantly low (11.1%; OR = 7.08, 95% CI [1.54–32.44]) in warthogs from the same area. In contrast to wild ruminants, seroprevalence was relatively low in sheep and goats from Namwala (4.8%; 95% CI [1.9–11.6]), Livingstone (4.2%; 95% CI [0.7–20.2]) and Mumbwa (17.5%; 95% CI, [10.0–28.6]) districts. No antibodies to RVFV were detected in goats and sheep from Kalomo and Namwala districts. Screening for RVFV genome in pooled mosquito (Table 2) samples did not yield positive results on Pan-Phlebo RT-PCR assay while all *Aedes* mosquito pools were negative for the RVFV genome on nested RT-PCR assay. Similarly, no RVFV genome could be detected in serum (*n* = 276; Table 1) samples on real-time RT-PCR assay [57].

**Table 4:** RVF seroprevalence in wild and domestic ruminants from Zambia.

Species	Province	District	Year	Season	No. Samples	Prevalence^†^
Buffalo	Central	Kabwe	2018	Dry	17	6 (35.3, 17.3–58.7)
	Central	KNP	2018	Dry	52	2 (3.9, 1.1–13.0)
	North-Western	Ikelenge	2016	Wet	29	7 (24.1, 12.2–42.1)
	Southern	MNP	2017	Dry	30	15 (50, 33.2–66.9)
Impala	Central	KNP	2017	Dry	98	46 (46.9, 37.4–56.8)
Warthog	Central	KNP	2017	Dry	18	2 (11.1, 3.1–32.8)
Hartebeest	Central	KNP	2017	Dry	41	18 (43.9, 29.9–59.0)
Sub-total^a^					285	96 (33.7, 28.5–39.4)
Goats	Southern	Namwala	2018	Dry	84	4 (4.8, 1.9–11.6)
	Southern	Livingstone	2018	Dry	24	1 (4.2, 0.7–20.2)
	Southern	Kalomo	2018	Dry	88	0/88
Sheep	Southern	Namwala	2018	Dry	13	0/13
Goats	Central	Mumbwa	2018	Dry	63	11 (17.5, 10.0–28.6)
Sub-total^b^					272	16 (5.9, 3.7–9.3)
Total					557	112 (20.1, 17.0–23.6)

^†^Positive (%, 95% CI); %, percent; CI, confidence interval

^a^Wildlife sub-total

^b^Domestic ruminants sub-total; KNP, Kafue National Park; MNP, Mosi-oa-Tunya National Park

### Past RVF outbreaks and La Niña events

**Fig 2:**
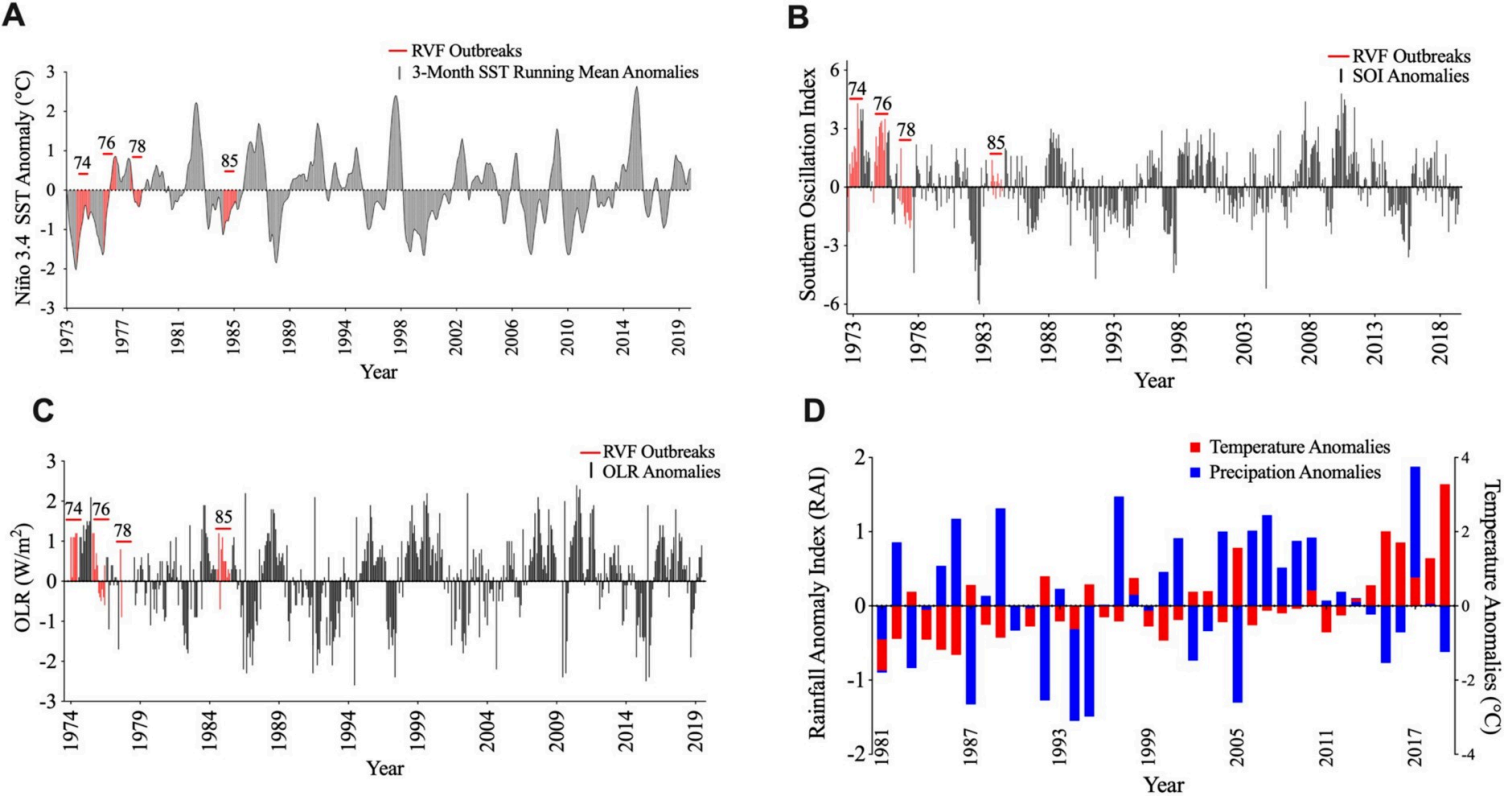
El Niño-Southern Oscillation indices. (A) Equatorial pacific (Niño 3.4) sea surface temperature anomalies (1973–2019). (B) Southern Oscillation Index (SOI) anomalies (1973–2019). (C) Outgoing long wave radiation anomalies (1974–2019). (D) Precipitation anomalies for Zambia (1981–2019) indicating wet (0–2) to extremely wet (>2) conditions.

There was a positive correlation between past RVF outbreaks (1974, 1976, 1985) and La Niña episodes, indicated by negative (< -0.5) 3-month running mean Niño 3.4 SST anomaly (Fig 2A). Similarly, positive SOI (> 1.0; Fig 2B) and OLR (> 1.0 W/m^2^; Fig 2C) anomalies were indicative of La Niña conditions prevailing in the equatorial Pacific Ocean. However, Niño 3.4 SST anomaly for 1978 was suggestive of neutral ENSO phase. Furthermore, precipitation anomalies for the period 1981–2019 indicated periods of anomalous wet conditions (1989, 1997, 2004, 2006, 2007, 2017; rainfall anomaly index (RAI) > 1) in the absence of RVF outbreaks (Fig 2D).

**Fig 3:**
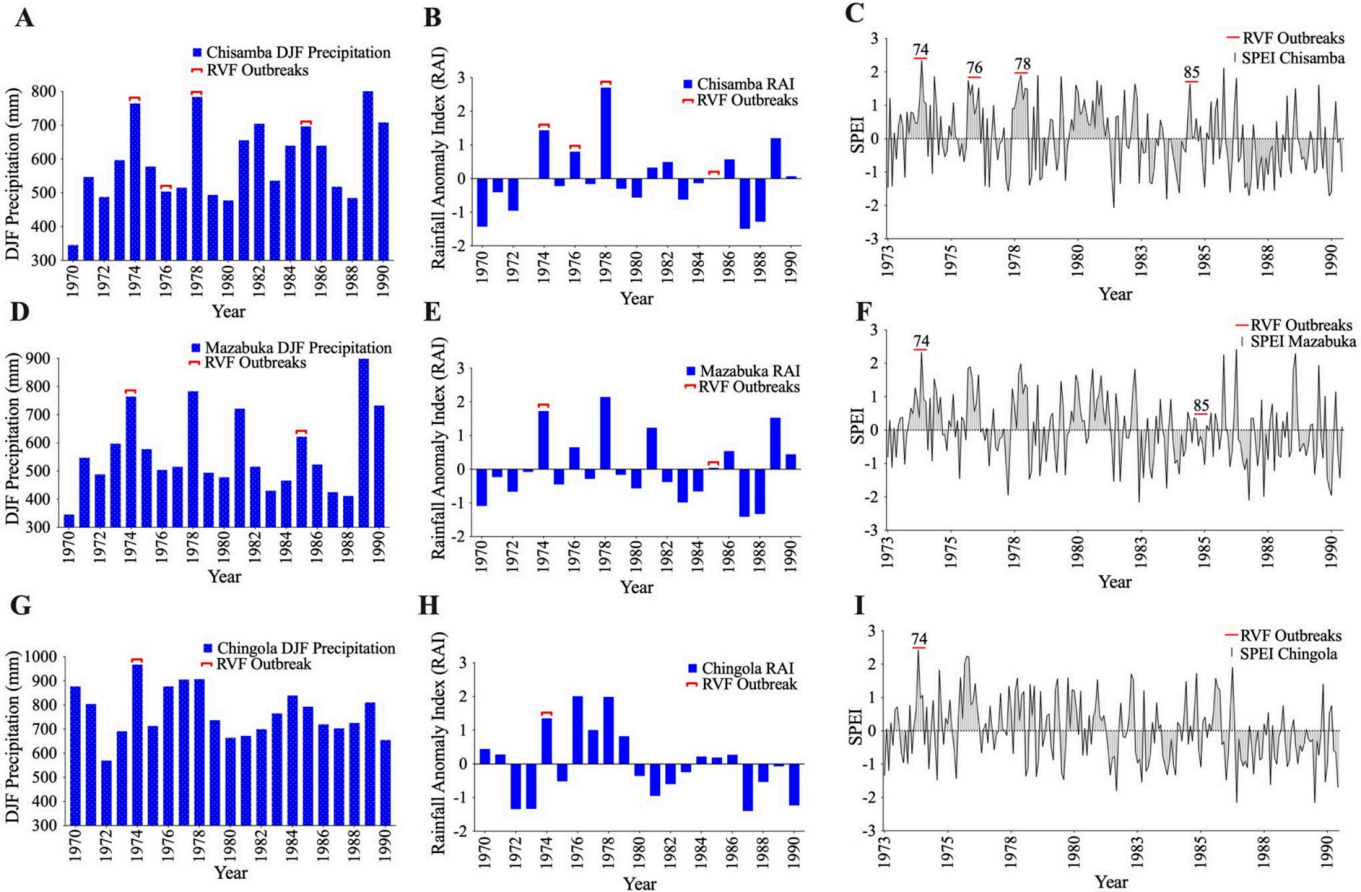
December-January-February (DJF) precipitation, rainfall anomaly index (RAI) and Standardized precipitation evapotranspiration index (SPEI) time-series analysis. (A,D,G) December-January-February precipitation during RVF outbreaks. (B,E,H) Rainfall anomaly Index indicating positive rainfall anomalies during RVF outbreaks. (C,F,I) Standardized precipitation evapotranspiration index (1973–1990) showing near normal, (-0.99–0.99), very wet (1.50–1.99) and extremely wet (>2.0) conditions during RVF outbreaks.

Likewise, analysis of DJF precipitation for Chisamba, Mazabuka, and Chingola districts showed three-months consecutive rainfall in excess of 500 mm during the 1974–1985 RVF outbreaks (Fig 2A, 2D and 2G). Also, positive rainfall anomalies (RAI > 0) were recorded during this period (Fig 2B, 2E and 2H), indicating normal to above normal rainfall. Time-series analysis of SPEI datasets (January 1, 1973 to December 31^st^, 1990) indicated near normal (-0.99–0.99) to extremely wet (> 2.00) conditions during RVF outbreaks. In Chisamba District, RVF outbreaks were indicated by moderately wet (1.00–1.49) to extremely wet conditions (> 2.00; Fig 3C). RVF outbreaks in Mazabuka (Fig 3F) and Chingola (Fig 3I) in 1974 were associated with extremely wet conditions (> 2.00) while the 1985 outbreak in Mazabuka was characterized by near normal conditions (-0.99–0.99).

### RVF risk Map

#### Mean NDVI

**Fig 4:**
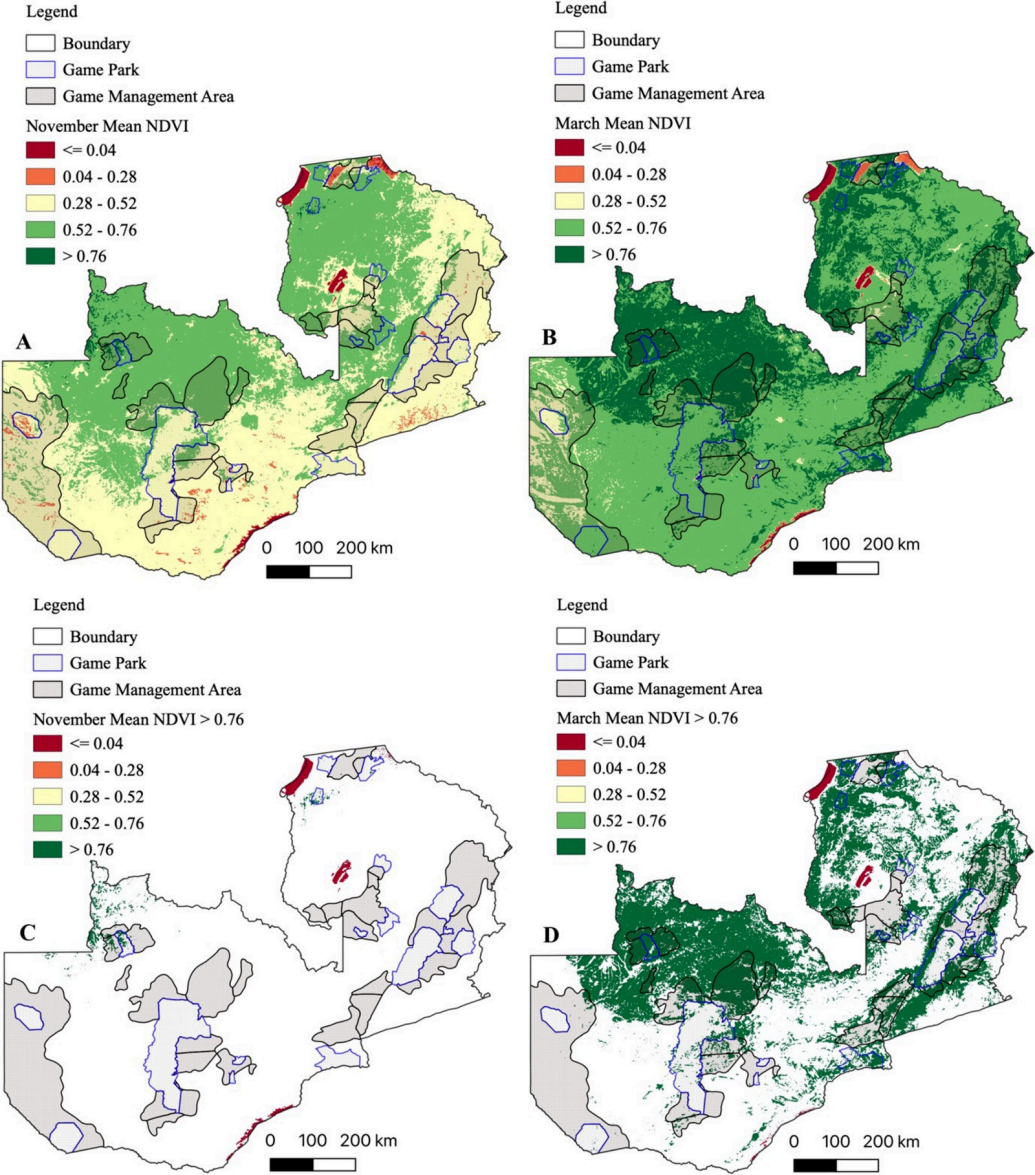
Mean normalized difference vegetation index (NDVI) for November and March. NDVI were computed as means for the period 2000–2019. (A & C) NDVI for November at the onset of the rainy season. (B & D) NDVI for March at the end of the rainy season showing anomalous (NDVI > 0.76) vegetation growth. Shapefile republished from DIVA-GIS database (https://www.diva-gis.org/) under a CC BY license of Global Administrative Areas (GADM), copyright 2018.

Satellite derived mean NDVI (2000–2019) showed spatiotemporal variations in vegetation greenness (Fig 4A and 4B). Increased vegetation response to precipitation was evident from November (Fig 4A–4C) through March (Fig 4B–4D). At the end of the rainy season in March, anomalous vegetation growth (NDVI > 0.76) was evident in much of north-western, northern and eastern parts of the country (Fig 4B–4D), however, the rest of the areas had sparse (NDVI < 0.76) vegetation cover (Fig 4B–4D).

**Fig 5:**
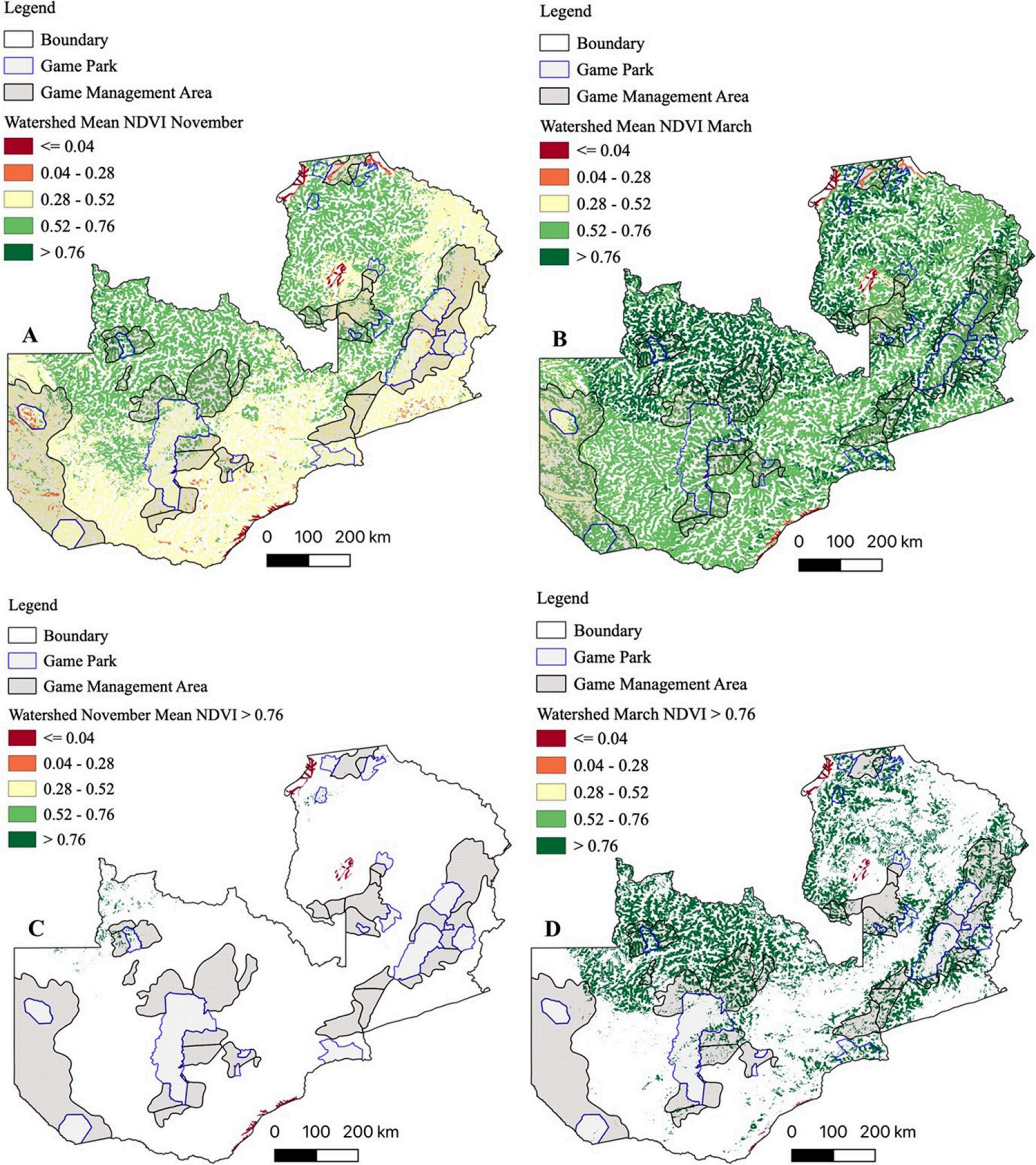
Riparian mean normalized difference vegetation index (NDVI) for the November and March. NDVI were calculated as means for the period 2000–2019. (A) Riparian NDVI for November. (B) Riparian NDVI for March. (C) Riparian NDVI > 0.76 for November showing RVF high risk areas. (D) Riparian NDVI > 0.76 for March showing RVF high risk areas. Shapefile republished from DIVA-GIS database (https://www.diva-gis.org/) under a CC BY license of Global Administrative Areas (GADM), copyright 2018.

Similar to what we observed on mean NDVI (Fig 4), riparian NDVI showed increased vegetation response to precipitation from the dry (Fig 5A–5C) to wet (Fig 5B–5D) season in catchment areas in north-western, northern and eastern parts of the country.

#### DJF precipitation and flood-prone areas

**Fig 6:**
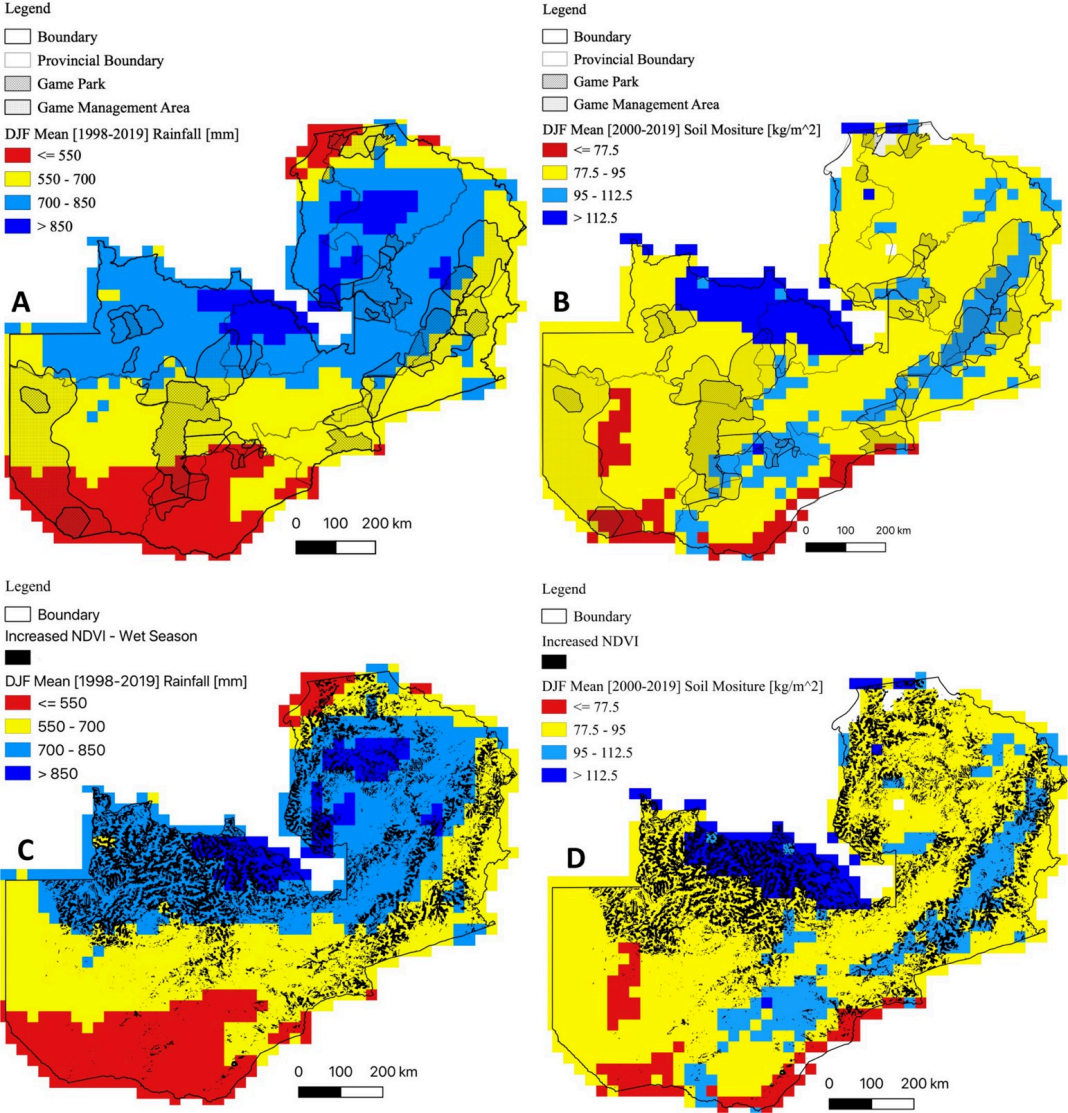
December-January-February (DJF) precipitation and soil moisture content. DJF precipitation and soil moisture content were computed as means with respect to the 1998–2019 and 2000–2019 climatological means, respectively. (A) Mean DJF precipitation showing high rainfall variability. (B) Mean DJF soil moisture content indicating areas that are at high risk of floods during seasons of above normal rainfall. (C) Correlation between increased riparian NDVI and mean DJF precipitation. (D) Correlation between increased riparian NDVI and mean DJF soil moisture content. Shapefile republished from DIVA-GIS database (https://www.diva-gis.org/) under a CC BY license of Global Administrative Areas (GADM), copyright 2018.

Mean DJF precipitation (1998–2019) showed high rainfall (> 700 mm) in north-western, northern and eastern parts of the country (Fig 6A). Highest DJF precipitation (> 850 mm) was observed in north-western and northern parts of the country. However, much of the southern, western and eastern parts of the country recorded low (< 700 mm) DJF rainfall. Equally, we noted a positive correlation between the spatial variations in DJF precipitation and increased riparian NDVI (Fig 6C). Areas with dense vegetation (NDVI > 0.76) recorded a high (> 700 mm) DJF precipitation. Furthermore, high DJF soil moisture (> 112.5 kg/m^2^) was observed in north-western, eastern and southern parts of the country (Fig 6B). Similarly, there was a positive correlation between high DJF soil moisture content and increased riparian vegetation (Fig 6D). The high DJF soil moisture content was indicative of likelihood of flooding in these areas.

#### Animal population density and RVF risk mapping

**Fig 7:**
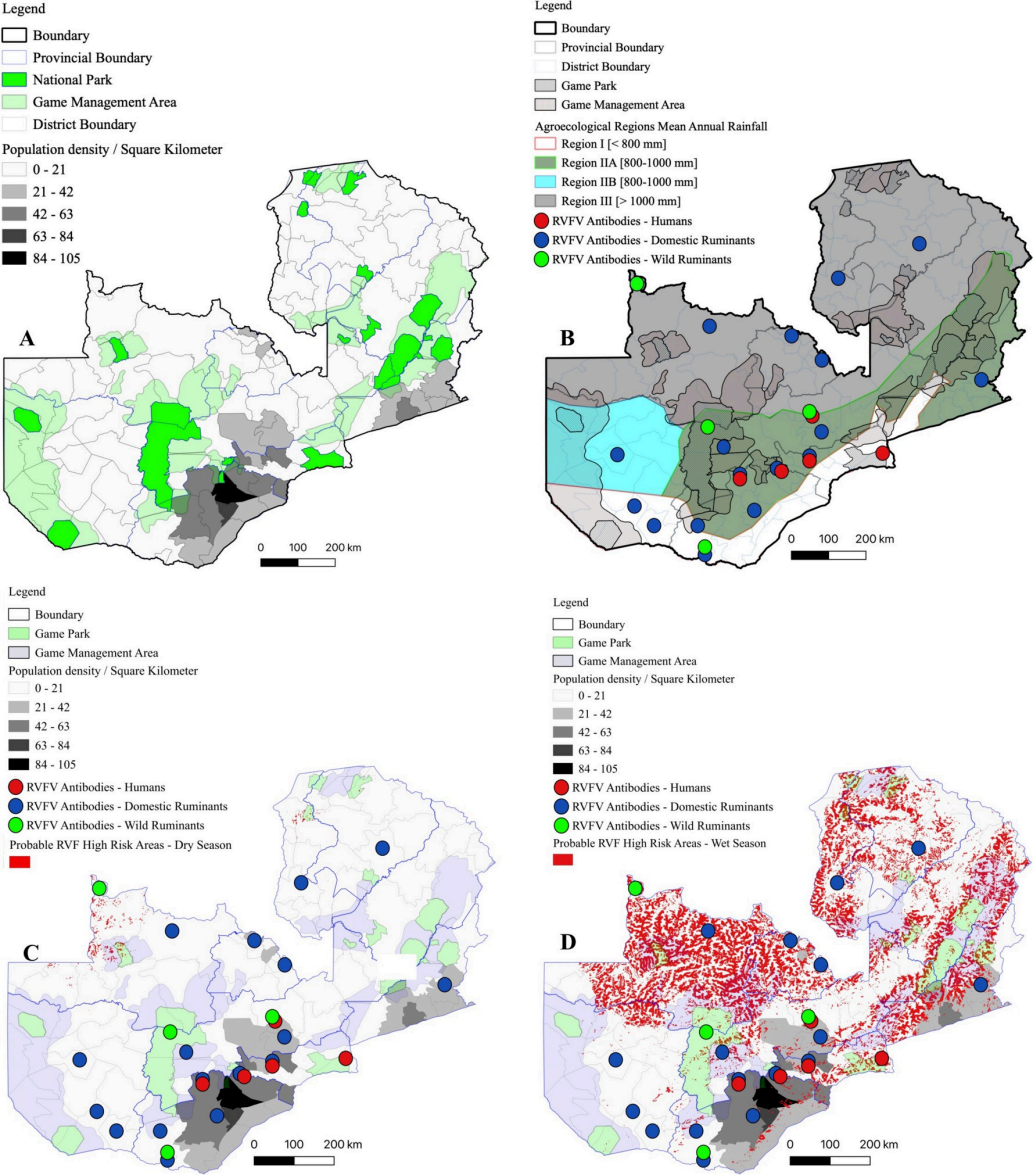
Ruminant population density and RVF risk map. (A) Domestic ruminant population density map. (B) Location of RVF outbreaks, past and present seropositive results. (C & D) RVF high risk areas in at the onset (November) and end of the rain season (March). Shapefile republished from DIVA-GIS database (https://www.diva-gis.org/) under a CC BY license of Global Administrative Areas (GADM), copyright 2018.

Population density for cattle, sheep and goats were combined to produce a single population density map for domestic ruminants (Fig 7A). Ruminant population was variable among districts. High ruminant population density (>21 animals/km^2^) was concentrated in the southern and eastern parts of the country. Nonetheless, these areas had poor DJF precipitation (< 700 mm; Fig 6C) while the soil moisture content (< 95 kg/m^2^; Fig 6D) was suggestive of low likelihood of flooding. Besides Chingola and Chililabombwe districts on the Copperbelt Province, there was a negative correlation between domestic ruminant population density, DJF rainfall and soil moisture content. Intriguingly, analysis of serosurveillance data from this and other studies indicated presence of RVFV antibodies in both low and high rainfall areas (Fig 7B), intimating presence of primary and secondary RVFV vectors. However, since RVF emergence is triggered by excessive rainfall and floods which result in the emergence of *Aedes Neomelaniconion* and *Aedimorphus* mosquitoes species [10,11], RVF high risk areas correspond to high rainfall and floods prone regions, which were also indicated by anomalous riparian vegetation growth at the end of the rain season in March (Fig 6C and 6D). Thus, by utilizing a combination of riparian NDVI > 0.76, mean DJF precipitation and soil moisture content, we mapped RVF risk areas during the dry (Fig 7C) and wet (Fig 7D) seasons in Zambia. The northern, north-western and eastern parts of the country were at high risk of RVF outbreaks although the domestic ruminant population density was low in these areas (Fig 7D). Conversely, despite the high ruminant population density, the southern parts of the country were at low risk of RVF emergence (Fig 7D).

#### Probable mosquito breeding habitats

**Fig 8:**
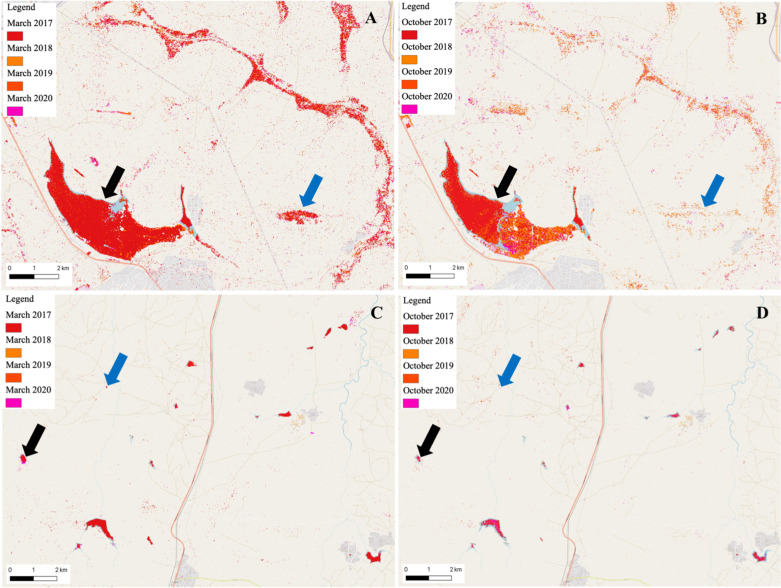
Permanent and ephemeral water bodies in Chililabombwe and Monze districts. Waterbodies were mapped as cumulative totals for March and October for the period 2017–2020. (A & B) Dambos in the Wet (March) and Dry Season (October) in Chililabombwe District on the Copperbelt Province. (C & D) Dambos in the wet (March) and dry (October) season in Monze District in Southern Province. Black and Blue arrows indicate permanent and ephemeral water bodies, respectively. Base map republished from OpenStreetMap (https://www.openstreetmap.org/copyright) under a CC BY license.

To account for the observed RVFV activity in both high and low risk areas (Fig 7D), we utilized sentinel-1 GRD datasets to detect permanent and ephemeral water bodies in Chililabombwe (high risk area; Copperbelt Province) and Monze (Low risk area; Southern Province) districts (Figs 8 and S2). Temporal variations in the water bodies were observed between the dry and wet seasons. Permanent waterbodies persisted throughout the dry season in both districts, while ephemeral water bodies were evident only in the rainy season. In Chililabombwe district, waterbodies were mainly seasonal wetlands in dried river beds (Fig 8A and 8B), while in Monze district, they consisted mainly of seasonal and permanent dams in dried riverbeds (Fig 8C and 8D).

## Discussion

In contrast to secondary ‘bridge’ mosquitoes captured in Zambia (*n* = 16,747) during the study period (2014–2019), only a limited number of *Neomelaniconion* (*n* = 31; *Aedes mcintoshi*) and *Aedimorphus* (*n* = 1; *Aedes ochraceus*) mosquitoes were caught in Mongu and Livingstone districts. While both districts were predicted to be at low risk of RVF outbreaks, the presence of seasonally inundated wetlands in the upper Zambezi River Basin [76] in Mongu District and the occurrence of dambos in Southern Province [77] (Fig 8C and 8D) could account for the presence of *Neomelaniconion* and *Aedimorphus* mosquitoes. However, the limited number of primary RVF vectors caught in both provinces during the wet season underscores the probable low risk of RVF emergence. Distinctly, our analysis indicated poor DJF rainfall and less likelihood of flooding (Fig 6A and 6B) in Southern and Western provinces. Nonetheless, the presence of secondary ‘bridge’ mosquitoes and the detection of RVFV seropositive animals in this and other studies [25,29] necessitates further investigation into the mechanisms of virus perpetuation and disease emergence in areas classified as ‘low risk’.

The absence of primary RVF vectors in high risk areas (Fig 7D), particularly in the north-western and northern parts of the country could be attributed to the fact that sampling was conducted only once at the onset (November) of the rainy season (S1 Table). Evidently, we observed seasonal variations in waterbodies in Chililabombwe District on the Copperbelt Province (Fig 8A and 8B). The reduction in volume of permanent water bodies and complete drying of ephemeral water bodies (Fig 8A and 8B) prior to the onset of the rainy season could be the likely reason for the lack of primary RVF vectors during the sampling period. Moreover, our analysis (Figs 6C, 6B and 7A) suggested that the ideal breeding period for primary RVF vectors was following sustained DJF rainfall (Fig 5D). Additionally, this and another previous study [25] demonstrated presence of RVFV antibodies in north-western and northern parts of the country, intimating likely presence of primary and secondary RVFV vectors in ‘high risk’ areas. To further clarify the presence of primary RVFV vectors and the risk of RVF emergence, more studies will need to be conducted in high risk areas particularly during the DJF period.

Even though it is widely reported that the RVFV is maintained *via* vertical transmission in *Neomelaniconion* and *Aedimorphus* mosquitoes, we could not detect RVFV genome in any Aedine mosquito samples. Whether this was due to the limited number of mosquitoes being screened or the lack of RVFV activity during the study period remains to be known. Even so, the high detection rate of RVFV seropositive animals during the inter-epizootic period along with the low detection rate in mosquitoes supports the hypothesis that horizontal, unlike vertical transmission, is important in pathogen maintenance [2]. Similarly, no RVFV genome could be detected in sera from wild and domestic ruminants. This finding was suggestive of lack of active infection or low virus activity during the interepizootic period. In a similar study in Tanzania [78], no RVFV genome could be detected in cattle during the interepizootic period. The low detection rate of RVFV during the interepizootic period is likely due to transient viraemia (< 7 days) in infected animals [79,80].

While there would be a potential for sampling bias, seroprevalence to RVFV antibodies was significantly high in wild ruminants compared to sheep and goats (33.3% vs 5.9%). Variations in seroprevalence rates in domestic and wild ruminants have been reported elsewhere in Africa [13]. In Southern Province, a perceived low risk area, seroprevalence was high (50.0%) in buffaloes from Mosi-oa-Tunya National park (Fig 1) compared to that in buffaloes from North Western Province (24.1%), a supposedly high risk area. Even though the reasons for this observation are not clear, the high number of secondary RVFV vectors caught in Southern Province during the study period (2014–2019) suggests potential for wide-spread RVFV infection. Intriguingly, we detected anti-RVFV antibodies in warthogs (11.1%; 2/18) and hartebeest (43.9%; 18/41) from Kafue National Park (Fig 1). RVFV antibodies have been previously reported in warthogs [67,81–83], however, there is limited information on the susceptibility of hartebeest to RVFV infection [84,85]. These findings highlight the probable wide host range of RVFV in Zambia and other sub-Saharan countries. Notably, due to the limited number of wildlife samples analyzed in this study (*n* = 285), the role of wildlife in the maintenance and transmission of RVFV in Zambia requires further clarification. Besides, there is no information linking wildlife, domestic ruminants, humans and mosquito vectors in the epidemiology of RVF in Zambia. Thus, more epidemiological studies are needed, particularly, at the wildlife-livestock-human interface areas in Zambia.

Analysis of past RVF outbreaks revealed a positive correlation between disease outbreaks and La Niña episodes, indicated by ENSO indices. The absence of RVF epizootics during some periods (Fig 2D; 1997, 2004, 2006, 2007, 2017) of anomalous rainfall (RAI > 1) is possibly due to the unique distribution of domestic ruminants in Zambia (Fig 7A). Notably, areas that were predicted to be at high risk of RVF epizootics had low ruminant population density while low risk areas had high ruminant population density (Fig 7D). In spite of this, the detection of RVF seropositive animals in both low and high risk areas (Fig 7D) requires further clarification on the transmission dynamics and mechanisms of virus perpetuation in the two ecological niches.

While this study raises important epidemiological aspects of RVF in Zambia, a number of limitations associated with the study can be improved upon in future. The limited number of serum samples collected during the study period and subsequent use of archived sera may have been a source of potential bias. Also, mosquito sampling was not uniform across regions, mainly due to poor accessibility of some areas during the rainy season. Analysis of past RVF outbreaks was solely based on ENSO indices, however, the influence of the ITCZ in the northern parts of the country was not taken into account.

In conclusion, even though RVF epizootics/-epidemics were last reported in 1985 in Zambia, our study suggests presence of, and enzootic circulation of RVFV in domestic and wild ruminants. This finding raises the potential for RVF emergence, particularly, in flood-prone, high rainfall areas in Zambia. We anticipate that this information will be used in planning surveillance and disease control programs in Zambia.

## Supporting information

S1 Table Number and composition of mosquitoes captured in Zambia between 2014 and 2019. (DOCX)

S1 Fig Detection of RVFV genome using Pan-Phlebo RT-PCR primers on 1.5% agarose gel. M, 100 bp DNA Marker; Yellow arrow, 500/517 bp mark; Lanes 1 (10^5^); 2 (10^4^); 3 (10^3^); 4 (10^2^); 5 (30); 6 (20); and 7 (10) represent RVFV genome L-segment copy number. Lane 8 was no template control.
(EPS)

S2 Fig Location of Chililabombwe and Monze districts in Zambia. Shapefile republished from DIVA-GIS database (https://www.diva-gis.org/) under a CC BY license of Global Administrative Areas (GADM), copyright 2018.
(EPS)
