# Supplementary material for: An unusually long Rift valley fever inter-epizootic period in Zambia: Evidence for enzootic virus circulation and risk for disease outbreak
Source: PLoS Negl Trop Dis. 2022 Jun 2;16(6):e0010420. doi: 10.1371/journal.pntd.0010420 (PMC9197056; doi:10.1371/journal.pntd.0010420)
Supplement: S1 Table — (DOCX) [file pntd.0010420.s001.docx]

**Table S1.** Mosquito surveillance data (2014-2019)

| **Province** | **District** | **Year** | **Month** | **Season** | **No. Mosquitoes** | **No. Pools** | ***Mosquito species*** |
| --- | --- | --- | --- | --- | --- | --- | --- |
| Southern | Siavonga | 2014 | April | Wet | 532 | 18 | *Culex quinquefasciatus* |
|  |  |  |  |  | 2 | 2 | *Culex sp.* |
|  |  |  |  |  | 76 | 5 | *Aedes aegypti* |
|  |  |  |  |  | 1 | 1 | *Aedes vittatus* |
|  |  |  |  |  | 1 | 1 | *Mansonia uniformis* |
|  |  |  |  |  | 1 | 1 | *Anopheles rufipes* |
|  |  |  |  |  | 1 | 1 | *Aedeomya sp.* |
|  |  | 2016 | March | Wet | 382 | 17 | *Culex quinquefasciatus* |
|  |  |  |  |  | 1 | 1 | *Culex cinereus* |
|  |  |  |  |  | 125 | 17 | *Aedes aegypti* |
|  |  |  |  |  | 6 | 4 | *Aedes sp.* |
|  |  |  |  |  | 2 | 2 | *Anopheles rufipes* |
|  |  |  |  |  | 1 | 1 | *Anopheles sp.* |
|  |  |  |  |  | 10 | 1 | *Aedes aegypti* |
|  |  |  |  |  | 2 | 1 | *Culex sp.* |
|  | Livingstone | 2015 | April | Wet | 773 | 26 | *Culex quinquefasciatus* |
|  |  |  |  |  | 1 | 1 | *Culex bitaenorhynchus* |
|  |  |  |  |  | 12 | 3 | *Aedes aegypti* |
|  |  |  |  |  | 8 | 1 | *Mansonia uniformis* |
|  |  |  |  |  | 2 | 1 | *Anopheles funestus* |
|  |  |  |  |  | 1 | 1 | *Anopheles coustani* |
|  |  |  |  |  | 1 | 1 | *Anopheles sp.* |
|  |  | 2017 | April | Wet | 5 | 5 | *Aedes aegypti* |
|  |  |  |  |  | 7 | 3 | *Aedes sp.* |
|  |  |  |  |  | 780 | 34 | *Culex quinquefasciatus* |
|  |  |  |  |  | 15 | 3 | *Culex univittatus* |
|  |  |  |  |  | 5 | 3 | *Culex nebulosus* |
|  |  |  |  |  | 11 | 3 | *Culex tigripes* |
|  |  |  |  |  | 1 | 1 | *Culex bitaenorhynchus* |
|  |  |  |  |  | 16 | 4 | *Anopheles funestus* |
|  |  |  |  |  | 8 | 4 | *Anopheles rufipes* |
|  |  |  |  |  | 9 | 1 | *Anopheles arabiensis* |
|  |  |  |  |  | 3 | 3 | *Anopheles coustani* |
|  |  |  |  |  | 34 | 1 | *Anopheles squamosus* |
|  |  |  |  |  | 2 | 2 | *Anopheles sp.* |
|  |  |  |  |  | 2 | 2 | *Mansonia sp.* |
|  |  |  |  |  | 1 | 1 | *Aedes sp.* |
|  |  | 2018 | Dec | Wet | 587 | 25 | *Culex quinquefasciatus* |
|  |  |  |  |  | 2 | 1 | *Aedes aegypti* |
|  |  |  |  |  | 1 | 1 | *Anopheles sp.* |
|  |  | 2019 | May | Wet | 1489 | 61 | *Culex sp.* |
|  |  |  |  |  | 65 | 5 | *Mansonia sp.* |
|  |  |  |  |  | 5 | 1 | *Anopheles sp* |
|  |  |  |  |  | 1 | 1 | *Aedes sp.* |
|  | Kazungula | 2015 | Dec | Wet | 45 | 5 | *Culex quinquefasciatus* |
|  |  |  |  |  | 4 | 2 | *Aedes aegypti* |
|  |  |  |  |  | 1 | 1 | *Aedes ochraceus* |
|  |  |  |  |  | 2 | 1 | *Mansonia uniformis* |
|  |  |  |  |  | 13 | 2 | *Anopheles funestus* |
|  |  |  |  |  | 8 | 2 | *Anopheles squamosus* |
|  |  |  |  |  | 8 | 1 | *Anopheles arabiensis* |
|  |  |  |  |  | 6 | 2 | *Anopheles coustani* |
|  |  |  |  |  | 3 | 2 | *Anopheles sp.* |
|  |  |  |  |  | 1 | 1 | *Aedeomya sp.* |
| Sub-total |  |  |  |  | 5081 (30.3%) | 292 |  |
| Lusaka | Lusaka | 2014 | April | Wet | 106 | 5 | *Culex quinquefasciatus* |
|  |  |  |  |  | 1 | 1 | *Culex sp.* |
|  |  |  |  |  | 3 | 1 | *Aedes aegypti* |
|  |  |  |  |  | 1 | 1 | *Anopheles funestus* |
|  |  | 2014 | June-July | Dry | 257 | 9 | *Culex quinquefasciatus* |
|  |  |  |  |  | 1 | 1 | *Culex trigripes* |
|  |  | 2015 | February | Wet | 440 | 15 | *Culex quinquefasciatus* |
|  |  |  |  |  | 2 | 1 | *Culex sp.* |
|  |  |  |  |  | 7 | 1 | *Aedes aegypti* |
|  |  |  |  |  | 1 | 1 | *Mansonia uniformis* |
|  |  |  | March | Wet | 1050 | 32 | *Culex quinquefasciatus* |
|  |  |  |  |  | 3 | 1 | *Culex trigripes* |
|  |  |  |  |  | 7 | 1 | *Culex sp.* |
|  |  |  |  |  | 13 | 2 | *Aedes aegypti* |
|  |  |  |  |  | 1 | 1 | *Mansonia uniformis* |
|  |  |  | June | Dry | 70 |  | *Culex quinquefasciatus* |
|  |  |  |  |  | 2 |  | *Culex sp.* |
|  |  |  | October | Dry | 1 | 1 | *Anopheles rufipes* |
| Sub-total |  |  |  |  | 1966 (11.7%) | 74 |  |
| Western | Mongu | 2014 | October | Dry | 1301 | 43 | *Culex quinquefasciatus* |
|  |  |  |  |  | 98 | 9 | *Culex sp.* |
|  |  |  |  |  | 26 | 6 | *Mansonia uniformis* |
|  |  |  |  |  | 19 | 2 | *Anopheles coustani* |
|  |  |  |  |  | 8 | 2 | *Coquillettidia fuscopennata* |
|  |  |  |  |  | 6 | 2 | *Coquillettidia metallica* |
|  |  |  |  |  | 5 | 2 | *Coquillettidia aurites* |
|  |  | 2016 | May | Wet | 278 | 15 | *Culex quinquefasciatus* |
|  |  |  |  |  | 126 | 5 | *Culex univittatus* |
|  |  |  |  |  | 9 | 3 | *Culex sp.* |
|  |  |  |  |  | 4 | 3 | *Aedes aegypti* |
|  |  |  |  |  | 99 | 5 | *Mansonia uniformis* |
|  |  |  |  |  | 182 | 9 | *Anopheles coustani* |
|  |  |  |  |  | 54 | 5 | *Anopheles squamosus* |
|  |  |  |  |  | 130 | 11 | *Anopheles sp.* |
|  |  |  |  |  | 121 | 7 | *Coquillettidia fuscopennata* |
|  |  |  |  |  | 32 | 3 | *Coquillettidia metallica* |
|  |  |  |  |  | 2 | 1 | *Uranotaenia balfouri* |
|  |  | 2017 | May | Wet | 285 | 22 | *Culex quinquefasciatus* |
|  |  |  |  |  | 309 | 15 | *Culex univittatus* |
|  |  |  |  |  | 41 | 5 | *Culex annulioris* |
|  |  |  |  |  | 2 | 2 | *Culex tigripes* |
|  |  |  |  |  | 109 | 5 | *Culex sp.* |
|  |  |  |  |  | 10 | 3 | *Aedes Mcintoshi* |
|  |  |  |  |  | 1 | 1 | *Aedes aegypti* |
|  |  |  |  |  | 9 | 2 | *Coquillettidia aurites* |
|  |  |  |  |  | 7 | 2 | *Coquillettidia metallica* |
|  |  |  |  |  | 46 | 5 | *Coquillettidia fusco* |
|  |  |  |  |  | 132 | 8 | *Mansonia sp.* |
|  |  |  |  |  | 1 | 1 | *Uranotaenia sp.* |
|  |  |  |  |  | 10 | 2 | *Aedeomya sp.* |
|  |  |  |  |  | 709 | 29 | *Anopheles coustani* |
|  |  |  |  |  | 173 | 8 | *Anopheles squamosus* |
|  |  |  |  |  | 7 | 4 | *Anophelesarabiensis* |
|  |  |  |  |  | 555 | 24 | *Anopheles sp.* |
|  |  | 2018 | August | Dry | 220 | 10 | *Culex quinquefasciatus* |
|  |  |  |  |  | 92 | 8 | *Culex univittatus* |
|  |  |  |  |  | 2 | 1 | *Culex tigripes* |
|  |  |  |  |  | 2 | 1 | *Anopheles coustani* |
|  |  |  |  |  | 1 | 1 | *Coquillettidia metallica* |
|  |  |  | December | Wet | 787 | 31 | *Culex quinquefasciatus* |
|  |  |  |  |  | 22 | 2 | *Culex univittatus* |
|  |  |  |  |  | 65 | 6 | *Culex sp.* |
|  |  |  |  |  | 4 | 1 | *Aedes Michintoshi* |
|  |  |  |  |  | 1 | 1 | *Aedes aegypti* |
|  |  |  |  |  | 1 | 1 | *Aedes sp.* |
|  |  |  |  |  | 59 | 4 | *Coquillettidia aurites* |
|  |  |  |  |  | 11 | 2 | *Coquillettidia metallica* |
|  |  |  |  |  | 32 | 5 | *Coquillettidia fuscopteron* |
|  |  |  |  |  | 392 | 16 | *Mansonia sp.* |
|  |  |  |  |  | 3 | 1 | *Uranotaenia sp.* |
|  |  |  |  |  | 1 | 1 | *Aedeomya sp.* |
|  |  |  |  |  | 33 | 2 | *Anopheles coustani* |
|  |  |  |  |  | 19 | 2 | *Anopheles squamosus* |
|  |  |  |  |  | 4 | 3 | *Anopheles sp.* |
|  |  | 2019 | May | Wet | 824 | 30 | *Culex quinquefasciatus* |
|  |  |  |  |  | 23 | 1 | *Culex univittatus* |
|  |  |  |  |  | 24 | 6 | *Culex sp.* |
|  |  |  |  |  | 17 | 3 | *Aedes Michintoshi* |
|  |  |  |  |  | 4 | 4 | *Aedes aegypti* |
|  |  |  |  |  | 15 | 4 | *Coquillettidia aurites* |
|  |  |  |  |  | 5 | 3 | *Coquillettidia metallica* |
|  |  |  |  |  | 60 | 4 | *Coquillettidia fusco* |
|  |  |  |  |  | 174 | 9 | *Mansonia sp.* |
|  |  |  |  |  | 1 | 1 | *Uranotaenia sp.* |
|  |  |  |  |  | 1 | 1 | *Aedeomya sp.* |
|  |  |  |  |  | 300 | 13 | *Anopheles coustani sp.* |
|  |  |  |  |  | 73 | 5 | *Anopheles squamosus sp.* |
|  |  |  |  |  | 165 | 8 | *Anopheles sp.* |
|  | Seheke | 2018 | December | Wet | 79 | 5 | *Culex quinquefasciatus* |
|  |  |  |  |  | 1 | 1 | *Aedes aegypti* |
| Sub-total |  |  |  |  | 8423 (50.2%) | 468 |  |
| Eastern | Chipata | 2015 | November | Wet | 286 | 12 | *Culex quinquefasciatus* |
|  |  |  |  |  | 19 | 3 | *Culex univittatus* |
|  |  |  |  |  | 1 | 1 | *Culex trigripes* |
|  |  |  |  |  | 1 | 1 | *Aedes aegypti* |
| Sub-total |  |  |  |  | 307 (1.8%) | 17 |  |
| Copperbelt | Kitwe | 2016 | November | Wet | 275 | 10 | *Culex quinquefasciatus* |
|  | Ndola |  |  |  | 15 | 1 | *Culex quinquefasciatus* |
|  |  |  |  |  | 4 | 1 | *Culex univittatus* |
| Sub-total |  |  |  |  | 294 (1.8%) | 12 |  |
| NorthWestern | Mwinilunga | 2016 | November | Wet | 37 | 5 | *Culex quinquefasciatus* |
|  |  |  |  |  | 3 | 1 | *Culex sp.* |
|  |  |  |  |  | 3 | 3 | *Aedes aegypti* |
|  |  |  |  |  | 1 | 1 | *Aedes vittatus* |
|  |  |  |  |  | 2 | 1 | *Anopheles funestus* |
|  |  |  |  |  | 1 | 1 | *Anopheles coustani* |
|  |  |  |  |  | 2 | 1 | *Uranotaenia balfouri* |
| Sub-total |  |  |  |  | 49 (0.3%) | 13 |  |
| Northern | Mpika | 2017 | November | Wet | 7 | 2 | *Culex quinquefasciatus* |
|  |  |  |  |  | 1 | 1 | *Aedes sp.* |
|  | Isoka | 2017 | November | Wet | 97 | 9 | *Culex quinquefasciatus* |
|  |  |  |  |  | 10 | 2 | *Culex univittatus* |
|  |  |  |  |  | 9 | 3 | *Culex bitaeno* |
|  |  |  |  |  | 4 | 1 | *Culex poicilipes* |
|  |  |  |  |  | 2 | 1 | *Culex perexigus* |
|  |  |  |  |  | 1 | 1 | *Culex sp.* |
|  |  |  |  |  | 2 | 2 | *Aedes aegypti* |
|  |  |  |  |  | 3 | 3 | *Aedes sp.* |
|  |  |  |  |  | 24 | 4 | *Coquillettidia aurea* |
|  |  |  |  |  | 68 | 6 | *Coquillettidia fuscopenata* |
|  |  |  |  |  | 15 | 2 | *Coquillettidia sp.* |
|  |  |  |  |  | 1 | 1 | *Mansonia sp.* |
|  |  |  |  |  | 5 | 1 | *Uranotaenia sp.* |
|  |  |  |  |  | 7 | 2 | *Anopheles funestus* |
|  |  |  |  |  | 2 | 2 | *Anopheles squamosus* |
|  |  |  |  |  | 5 | 3 | *Anopheles arabiensis* |
|  |  |  |  |  | 12 | 4 | *Anopheles sp.* |
|  | Mpulungu | 2017 | November | Wet | 359 | 20 | *Culex quinquefasciatus* |
|  |  |  |  |  | 6 | 4 | *Culex univittatus* |
|  |  |  |  |  | 1 | 1 | *Culex bitaeno* |
|  |  |  |  |  | 1 | 1 | *Culex tigripes* |
|  |  |  |  |  | 8 | 5 | *Aedes aegypti* |
|  |  |  |  |  | 4 | 2 | *Aedes sp.* |
|  |  |  |  |  | 1 | 1 | *Mansonia sp.* |
|  |  |  |  |  | 2 | 1 | *Anopheles arabiensis* |
|  |  |  |  |  | 1 | 1 | *Anopheles rufipes* |
| Sub-total |  |  |  |  | 658 (3.9%) | 86 |  |
| Total |  |  |  |  | 16778 | 961 |  |
